# Prevalence and predictors of postpartum depression among postnatal women in Lagos, Nigeria

**DOI:** 10.4314/ahs.v20i4.53

**Published:** 2020-12

**Authors:** EO Adeyemo, EO Oluwole, OJ Kanma-Okafor, OM Izuka, KA Odeyemi

**Affiliations:** 1 Department of Community Health and Primary Care, College of Medicine University of Lagos. Lagos State, Nigeria; 2 Federal Medical Centre Umuahia, Abia State, Nigeria

**Keywords:** Postpartum depression, PPD, Eti-Osa, postnatal women, Lagos

## Abstract

**Background:**

Globally, postpartum depression is one of the most common but often unrecognized complications of childbirth, yearly affecting about 10–15% of postnatal women. This study aimed to determine the prevalence of postpartum depression and its predictors among postnatal women in Lagos.

**Methods:**

A descriptive cross-sectional study was conducted among 250 mothers in Eti-Osa Local Government Area of Lagos State, Nigeria, attending six Primary Health Care centers for infant immunization at six weeks post-delivery. Data was collected using a pretested semi-structured interviewer administered questionnaire which included the Edinburgh Postnatal Depression Scale. Analysis was carried out using SPSS version 23TM. Chi-square and logistic regression analyses were used to determine associations and predictive relationships between various factors and the presence of postpartum depression. The level of significance was set at <0.05.

**Results:**

The prevalence of postpartum depression was 35.6%. Multiparity, delivery by cesarean section, mother being unwell after delivery, and not exclusively breastfeeding the baby were the factors linked with postpartum depression. Following multiple logistic regression, having postpartum blues (p=0.000; OR=32.77; 95%CI=7.23–148.58)., not getting help with caring for the baby (p=0.008; OR=2.64; 95%CI=1.29–5.42), experiencing intimate partner violence (p=0.000; OR=5.2; 95%CI=2.23–11.91) and having an unsupportive partner (p=0.018; OR=2.6; 95%CI=1.17–5.78) were identified as predictors of postpartum depression.

**Conclusion:**

This study revealed a high prevalence of postpartum depression, identifying both the obstetric and psychosocial predictors. Social support for women both in the pre- and postnatal periods and routine screening of women for postpartum depression should be encouraged for early detection and immediate intervention.

## Introduction

Postpartum depression (PPD) is a mental health disorder that yearly affects about 10–15% of mothers worldwide.^1^ It sets in immediately or about two to six weeks after delivery and may last for over a year.^2^ It is characterized by symptoms such as tearfulness, a feeling of hopelessness, emotional lability, feelings of guilt, sleep problems and loss of appetite.^2^, ^3^ As joyful and as exciting as the birth of a baby can be to a mother, it can be emotionally draining, tasking, and stressful leading to a depressed mood which affects a woman's quality of life, social functioning and economic productivity.^4^

PPD has a significant impact on the mother and longterm consequences on the cognitive and emotional development of most children whose mothers are affected.^5^ It is generally also agreed that while this illness can progress into major depression and carries a great risk of ill health and death, it is an underdiagnosed and underrated illness in many countries. In addition, it has been reported that the prevalence of PPD is three times higher in developing countries compared to developed countries, with various risk factors accounting for the high burden of the illness.^6^ The estimated prevalence of PPD in Africa is 18.4%. However, various countries have reported higher rates such as Uganda (43.0%) and Cameroun (23.4%) as compared to Ethiopia (13.1%), Ghana (3.8%) and Morocco (11.6%).^7^,^8^,^9^,^10^ In Nigeria, various studies have been conducted to determine the prevalence of PPD using the Edinburgh Postnatal Depression Scale (EPDS). In western Nigeria, the lowest and highest prevalence of PPD reported were 14.6% and 23.0% respectively.^11^,^12^ Two different studies conducted in South-eastern Nigeria reported a low prevalence of 10.7% in one and a high prevalence of 30.0% in the other.^13^,^14^ In Northern Nigeria, seemingly high prevalence rates of 44.5% and 21.8% were reported.^15^,^16^ The varying prevalence worldwide and within Nigeria could be attributed to the different types of screening methods used, the study designs, the differences in geographical location, differences in socioeconomic status, the cut off score of the screening instruments as well as the various risk and predictive factors associated with developing PPD in such studies.

Socio-demographic factors such as religion, age, socioeconomic status, education and unemployment have been linked with PPD.^17^,^18^ Also, obstetric and infant care factors such as unplanned pregnancy, complications in pregnancy, an unhealthy baby, the death of an infant, parity, and history of abortion, have been reported as risk factors.^19^ In addition, other risk factors such as lack of antenatal care, history of psychiatric illness, stress, marital problems, and lack of emotional or social support have also been identified.^20^ Among all, the most stable risk factors/predictors reported over time include a prior history of depression, inadequate social support, poor quality of the mother's relationship with her partner, and stress.^21^

In Nigeria, certain factors may serve as deterrents to PPD. The communal-living lifestyle found especially in rural settings enables social support and companionship from members within the community. This gives a sense of comfort and relief to women, from the challenges of pregnancy and delivery. In addition, the traditional naming ceremony that is typically celebrated on the baby's eighth day of life in some African cultures, enables mothers to maintain high spirits in the first few days after delivery. Furthermore, the cultural practice of “omugwo/ olojojo omo” where a woman's mother and/or her mother in-law takes residence with her after her delivery for periods up to 6 months, to care for both mother and baby, helps the newly delivered woman to adjust to life after childbirth. These practices reduce the stress and anxiety that come with motherhood and hence limiting the risk of developing PPD. However, certain factors act as enablers of PPD. Many women during postnatal visits to the clinic may conceal their emotions and view PPD as normal rather than as an illness of concern, thus may keep their feelings to themselves, bottling up in silence.^22^ Furthermore, many women are unaware of the signs and symptoms of the illness and those that are aware that they have a problem, tend to keep quiet about it for fear of being stigmatized or considered weak. In Nigeria, there is poor knowledge of PPD among postnatal women and poor recognition of its symptoms by health practitioners.^23^ This has resulted in missed diagnosis of PPD and has necessitated research on the prevalence and associated risk factors of PPD to provide evidence of the burden of PPD. Though few studies have been carried out on the prevalence of PPD and its risk factors in many tertiary hospitals in Nigeria, there is still a paucity of work done on PPD among women, particularly at 6 weeks postpartum. Thus, this study was aimed at determining the prevalence, risk factors and predictors of PPD among postnatal women, six weeks after delivery.

## Methods

A descriptive cross-sectional study was carried out in six primary healthcare centres (PHCs) in Eti-Osa Local Government Area (LGA) in Lagos, Nigeria among women, 6 weeks postpartum, attending infant immunization clinics. The minimum sample size of 250 was determined using the Cochran formula, 24 with a standard normal deviate at 95% confidence interval of 1.96, a prevalence rate of 0.14610 and the error of precision at 5%, after adjusting for non-response. The participants were selected by the multistage sampling technique. In stage one Eti-Osa LGA was selected out of 20 LGAs in Lagos by simple random sampling using balloting. In stage two, 6 out of 16 administrative wards of the LGA were selected by balloting. Stage three involved the selection of 1 PHC in each of the 6 selected wards, also by balloting. In stage four, a maximum of 41 study participants were consecutively recruited at each PHC over a period of 2 months from June to August 2018.

Data were collected using a pretested, semi-structured, interviewer-administered questionnaire which was divided into three sections. The first section was to collect data on the socio-demographic characteristics of the respondents; the second section included the obstetric, child-related and psychosocial risk factors of PPD while the third section consisted of the Edinburg PPD Scale (EPDS), a 10-question screening tool for PPD.^25^ Each question in the EPDS had 4 answers scored 0, 1, 2 or 3. To determine the prevalence of PPD, all the scores were summed up. The minimum and maximum total scores obtained from the EPDS were 0 and 30 respectively. An EPDS score ≥13 was considered positive for PPD, while a score of<13 ruled out the possibility of PPD.^25^

Data analysis was carried out with SPSS version 23.0™. Chi-square and logistic regression analyses were used to determine associations and predict the relationships between the dependent variable (PPD) and socio-demographic and other risk factors. A p-value of p<0.05 was considered statistically significant. Ethical approval was obtained from the Lagos University Teaching Hospital (LUTH), Health Research Ethics Committee. Written informed consent was obtained from each participant before enrollment into the study. It was clearly explained that participation was purely voluntary with no penalty for non-participation and that participants could choose at any time to withdraw from the study. All the information collected were handled as strictly confidential.

## Results

The mean age of the respondents was 29.5±5.70 SD, with 57.6% within the age group 21 to 30yrs. A greater proportion of the respondents were Christians (62.4%), majority (91.6%) married or in-union, 85.2% of them being monogamous marriages. The Yoruba tribe made up 40.4%, while the Igbos were 23.2%, the Hausas 7.6%, other tribes (Tiv, Ibira, Efik) made up 28.8% of the respondents. The greatest proportion (37.6%) of the respondents were traders, 21.2% were housewives, 21.6% artisans and 12.8% were professionals. However, 6.8% of them were unemployed. Up to 20% of the mothers had a low family income of <₦ 10,000 monthly, though about half of the respondents had a family income of ₦ 10,001 to ₦ 50,000 monthly (Table 1).

**Table 1:** Socio-demographic characteristics of the respondents

Variable	Frequency (n=250)	Percent (%)
**Age**		
≤20years	12	4.8
21 – 30years	144	57.6
31 – 40years	87	34.8
≥ 41years	7	2.8
Mean age ±SD	**29.52±5.70**	
**Religion**		
Christian	156	62.4
Muslim	93	37.2
Others	1	0.4
**Marital Status**		
Single	12	4.8
Married/in-union	229	91.6
Separated/Widowed/Divorced	9	3.6
**Marriage/union type (n=229)**		
Monogamy	213	85.2
Polygamy	16	14.8
**Ethnicity**		
Yoruba	101	40.4
Igbo	58	23.2
Hausa/Fulani	19	7.6
Others	72	28.8
**Level of education**		
None formal	18	7.2
Primary	58	23.2
Secondary	124	49.6
Tertiary	50	20
**Occupation**		
Professional	32	12.8
Housewife	53	21.2
Artisans	54	21.6
Trader/Business	94	37.6
Unemployed	17	6.8
**Monthly family income (in Naira)**		
≤10,000	50	20.0
10,001 – 50,000	109	43.6
50,001 – 100,000	47	18.8
100,001 – 200,000	29	11.6
200,001 – 400,000	10	4.0
>400,000	5	2.0

The majority (74.0%) of the respondents did not experience any complications during pregnancy. More than half (61.2%) had 2 to 4 children. Vaginal delivery was the most common mode of delivery among respondents. Almost a third (26.6%) of the respondents had been unwell since their delivery. As high as 67.2% of the respondents had experienced postpartum blues, which had most frequently been attributed to lack of support in caring for the baby and themselves (38.1%), followed by having insufficient finances (30.4%). Male children were the desire of 42.0% of the respondents, though 47.6% eventually had male babies. An optimal birth weight (2.5kg – 4.0kg) was found with 87.6% of the babies, while 69.6% had optimal weights of >4kg to 6kg at six weeks of life. Majority of the babies were exclusively breastfed (68.8%) (Table 2).

**Table 2:** Obstetric and child-related characteristics of respondents

Variable	Frequency (n=250)	Percent (%)
**Problems or complications in pregnancy**		
Yes	65	26.0
No	185	74.0
**Parity**		
1	76	30.4
2–4	153	61.2
≥ 5	21	8.4
**Mode of last delivery**		
Vaginal	213	85.2
Caesarean section	37	14.8
**Mother's health since delivery**		
Unwell	67	26.8
Well	183	73.2
**Postpartum blues (PPB)**		
Present	168	67.2
Absent	82	32.8
**Supposed triggers of PPB (n=168)**		
No help or support in caring for baby	64	38.1
Stress	33	19.6
Death of a loved one	10	6.0
Insufficient funds	51	30.4
Others	10	6.0
**Desired sex of the baby**		
Male	105	42.0
Female	94	37.2
Not specific	51	20.4
**Actual sex of the baby**		
Male	119	47.6
Female	131	52.4
**Weight of baby at birth**		
<2.5kg	19	7.6
2.5–4.0kg	219	87.6
>4.0kg	12	4.8
**Weight of baby at 6 weeks**		
<4.0kg	56	22.4
>4.0–6.0kg	174	69.6
>6.0kg	20	8.0
**Method of feeding baby**		
Exclusive breast feeding	172	68.8
Mixed feedings	65	26.0
Formula feedings	13	5.2

A small proportion (9.2%) of the respondents had a family history of mental illness. While 82.8% had supportive husbands, 17.2% of the husbands were unsupportive by not providing enough financially (39.5%), not helping with the care of their children (32.6%), not giving care and not paying enough attention to the woman (27.9%). Husbands were violent towards 16% of the respondents; 27.5% of the respondents were beaten, 7.5% were raped and 65% verbally abused. Help for the care of the baby was most frequently given by relatives (39.2%) but 32.4% of the respondents did not get any help at all. Husbands were helpful in 20.4% of cases. (Table 3)

**Table 3:** Psychosocial characteristics of respondents

Variable	Frequency (n=250)	Percent(%)
**Family history of mental illness**		
Yes	23	9.2
No	227	90.8
**Husband/partner supportiveness**		
Yes	207	82.8
No	43	17.2
**Mode of unsupportiveness (n=43)**		
Not providing enough financially	17	39.5
Not helping out with the care of the	14	32.6
children		
Not giving care and attention	12	27.9
**Husband/partner violence**		
Yes	40	16.0
No	210	84.0
**Type of violence (n=40)**		
Beating	11	27.5
Rape	3	7.5
Verbal abuse	26	65.0
**Source of help for the care of the baby**		
Husband	51	20.4
Relatives	98	39.2
Friends	9	3.6
Nanny/House-help	11	4.4
None	81	32.4

**Figure 1:**
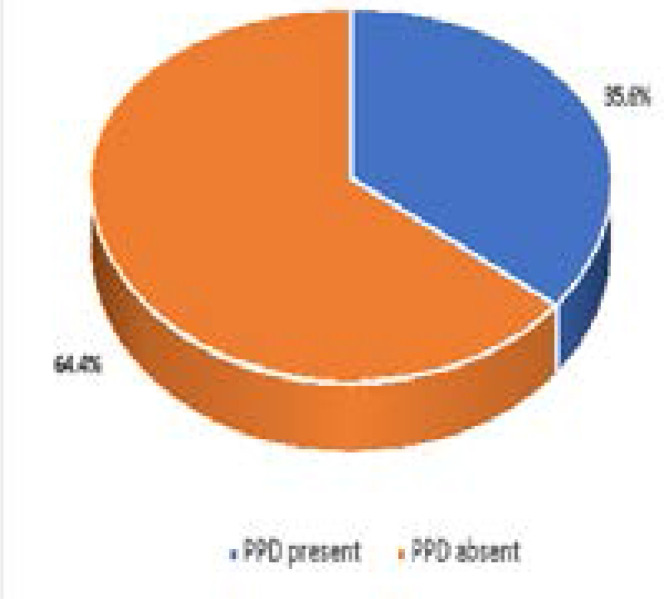
Prevalence of PPD

The prevalence of PPD was 36.5% (Figure 1). There was no statistically significant association between the socio-demographic characteristics of the participants and the presence of PPD (Table 4). Among the obstetric and child-related factors of the respondents, having more than five children (p=0.027), mode of delivery, by cesarean section (p=0.002), mothers' poor state of health since delivery (p<0.001), experiencing postpartum blues (p<0.001) and not exclusively breastfeeding the baby (p=0.003) were associated with PPD (p<0.05) (Table 5). Furthermore, some psychosocial factors were significantly associated with having PPD; having an unsupportive partner (p<0.001), experiencing intimate partner violence (p<0.001) and not getting help in taking care of their baby (p<0.001) (Table 6). After subjecting the associated factors to logistic regression analysis to eliminate confounders, postpartum blues, lack of assistance in taking care of the baby, intimate partner violence and having an unsupportive partner remained as predictors for developing PPD. Postpartum blues was identified as a predictor of PPD (p=0.000; OR=32.77; 95%CI=7.23–148.58). This implies that women who experienced postpartum blues within 0 – 2 weeks after delivery were likely to develop PPD, and the odds that women who experienced postpartum blues would get depressed was 32 times higher than those who did not experience postpartum blues. In addition, not having help after childbirth (p=0.008; OR=2.64; 95%CI=1.29–5.42) was also identified as a predictor of PPD such that women who did not have help in taking care of their babies were more than 2 times as likely to develop PPD compared to those who did. Experiencing intimate partner violence (p=0.000; OR=5.2; 95%CI=2.23–11.91) was implicated as a predictor of PPD, implying that women who experienced violence from their partners were about 5 times more likely to develop PPD than those who did not. Also, having an unsupportive partner (p=0.018; OR=2.6; 95%CI=1.17–5.78) was identified as a predictor of PPD, such that those mothers who had unsupportive partners were almost 3 times more likely to have PPD than those who received support from their partners. (Table 7).

**Table 4:** Socio-demographic factors associated with PPD

Variable	PPD present n=89 Freq(%)	PPD absent n=161 Freq(%)	Chis-quare	p-value
**Age**				
≤20years	2(16.7)	10(83.3)		0.477*
21 – 30years	51(35.4)	93(64.6)		
31 – 40years	34(39.1)	53(60.9)		
≥41years	2(28.6)	5(71.4)		
**Religion**				
Christian	58(37.2)	98(62.8)		0.296*
Muslim	30(32.3)	63(67.7)		
Others	1(100.0)	0(0.0)		
**Marital**				
Single	6(50.0)	6(50.0)		0.236*
Married/co-habiting	78(34.1)	151(65.9)		
Separated / Widowed /Divorced	5(55.6)	4(44.4)		
**Marriage type (n=229)**				
Monogamy	74(34.7)	139(65.3)	0.14	0.707
Polygamy	5(31.3)	11(68.8)		
**Ethnicity**				
Yoruba	28(27.7)	73(72.3)	6.94	0.074
Igbo	27(46.6)	31(53.4)		
Hausa/Fulani	9(47.4)	10(52.6)		
Others	25(34.7)	47(65.3)		
**Level of education**				
Non-formal	9(50.0)	9(50.0)		0.334*
Primary	23(39.7)	35(60.3)		
Secondary	41(33.1)	83(66.9)		
Tertiary	13(28.9)	32(71.1)		
Postgraduate	3(60.0)	2(40.0)		
**Occupation**				
Professional	14(43.8)	18(56.3)	7.19	0.126
Housewife	24(45.3)	29(54.7)		
Artisan	14(25.9)	40(74.1)		
Trade/Business	29(30.9)	65(69.1)		
Unemployed	8(47.1)	9(52.9)		
**Monthly family Income**				
≤10,000	19(38.0)	31(62.0)		0.267*
10,001 – 50,000	31(28.4)	78(71.6)		
50,001 – 100,000	18(38.3)	29(61.7)		
100,001 – 200,000	13(44.8)	16(55.2)		
200,001 – 400,000	6(60.0)	4(40.0)		
≥400,000	2((40.0)	3(60.0)		

* Fisher's exact p-value

**Table 5:** Obstetric and child-related factors associated with the presence of PPD

Variable	PPD present	PPD absent	X^2^	p-value
	n=89 Freq(%)	n=161 Freq(%)		
**Complications in pregnancy**				
Yes	26(40.0)	39(60.0)	0.74	0.389
No	63(34.1)	122(65.9)		
**Parity**				
1	31(40.8)	45(59.2)	7.19	**0.027**
2–4	46(30.1)	107(69.9)		
≥ 5	12(57.1)	9(42.9)		
**Mode of Delivery**				
Vaginal	67(31.5)	146(68.5)	10.78	**0.001**
Caesarean Section	22(59.5)	15(40.5)		
**Mother's health since delivery**				
Unwell/ill	35(52.2)	32(47.8)	13.14	**<0.001**
Well	54(29.5)	129(70.5)		
**Postpartum blues**				
Present	87(51.8)	81(48.2)		<0.001*
Absent	2(2.4)	80(97.6)		
**Desired sex of baby**				
Male	32(30.5)	73(69.5)	2.20	0.333
Female	37(39.8)	56(60.2)		
Not Specific	20(39.2)	31()60.8		
**Actual sex of baby**				
Male	39(32.8)	80(67.2)	0.79	0.374
Female	50(38.2)	81(61.8)		
**Babies weight at birth**				
<2.5kg	10(52.6)	9(47.4)		0.297*
2.5 – 4.0kg	75(34.3	144(65.8)		
>4.0kg	4(33.3)	8(66.7)		
**Babies weight at 6 weeks**				
2.01 – 4.00 kg	22(39.3)	34(60.7)	0.63	0.731
4.01 – 6.00 kg	61(35.1)	113(64.9)		
6.01 – 8.00kg	6(30.0)	14(70.0)		
**Feeding method**				
Exclusive Breast feeding	53(30.8)	119(69.2)		0.003*
Mixed feeding	26(40.0)	39(60.0)		
Formula feeding	10(76.9)	3(23.1)		

* Fisher's exact p-value

**Table 6:** Psychosocial factors associated with PPD

Variable	PPD present n=89(%)	PPD absent n=161(%)	X^2^	p-value
**Family history of mental illness**				
Yes	12(52.2)	11(47.8)	3.035	0.080
No	77(33.9)	150(66.1)		
**Husband/partner violence**				
Yes	30(75.0)	10(25.0)	32.243	**<0.001**
No	59(28.1)	151(71,9)		
**Help with caring for the baby**				
Present	42(24.9)	127(75.1)	30.359	**<0.001**
Absent	47(58.0)	34(42.0)		
**Supportiveness of husband**				
Yes	60(29.0)	147(71.0)	22.967	**<0.001**
No	29(67.4)	14(32.6)		

**Table 7:** Predictors of PPD

Variable	aOR	95% CI	p-value
Having an unsupportive partner	**2.6**	**1.17–5.78**	**0.018**
Experiencing intimate partner violence	**5.2**	**2.23–11.91**	**<0.001**
Not having help with caring for the baby	**3.2**	**1.71–5.79**	**<0.001**
Having postpartum blues	**32.8**	**7.23–148.58**	**<0.001**

aOR=adjusted odds ratio CI=confidence interval

## Discussion

The prevalence of PPD found in this study was 35.6%. This finding is similar to the prevalence ranges of 35–47%, 28–57%, and 35–47% for Latin America, Bangladesh and Pakistan respectively.^25^ Similarly, in other countries such as Jamaica and Korea, the prevalence of PPD reported at 6 weeks post-delivery were 34.6% and 34.3% respectively.^26^ In Africa likewise, a prevalence of 34.7% was reported in South Africa and 16%–34.2% in Zimbabwe.^27^,^28^ A study conducted in South Eastern Nigeria reported a prevalence of 30.6% at EPDS cut off score of 10.^29^ Also, a study in North-East Nigeria reported a prevalence of 44.5%, using a cut-off score of 7.^14^ On the other hand, some other studies in Nigeria reported much lower prevalence rates; Osun State (14.6% at EPDS cut off 8/9),^20^ Enugu (10.7% at the optimal EPDS cut-off score of 9),^13^ and Lagos (20.9% at EPDS cut-off of >12).^30^ The differences in prevalence from various studies compared to this study may be due to differences in the study designs, the postpartum period at which the study was conducted, the differences in geographical location (developed or developing countries), different risk factors studied, and the cut off score of the screening instrument.

In this study no association was found between socio-demographic characteristics of the respondents and development of PPD. This study corroborated a study conducted in Enugu, South-East Nigeria which reported that there was no association between PPD and socio-demographic characteristics of women 6 weeks after delivery.^31^ Similarly, a study conducted in Cameroon with a PPD prevalence of 23.4 %, reported that socio-demographic variables were not associated with developing PPD.^8^ However, a study in Iraq implicated low socio-economic profiles of respondents in PPD.^32^

In both economically developed and developing nations, unplanned pregnancy, mode of delivery (Caesarean section), high parity, premature delivery, unintended and unwanted pregnancy, infant illness after delivery and not breastfeeding have been reported as risk factors of PPD.^33^ In this study, the results revealed that various obstetric and child related factors were associated with PPD. Parity (p=0.027) was identified as a risk factor associated with PPD, which was in line with findings from other studies.^34^ In addition, it revealed that more of the women who had more than 5 children had PPD and this could be as a result of the stress that comes with having many children. Also, the mode of delivery (p=0.002) was implicated as a risk factor to having PPD with more of the women who had caesarean sections having PPD. Studies in United Arab Emirates,^35^ France,^36^ and Finland^37^ also found the mode of delivery to be a risk factor for PPD. Furthermore, a study in Greece and another in Nigeria reported a significant association between PPD and mode of delivery among women who delivered via Caesarean section.^38^ Experiencing a form of illness after delivery was also associated with PPD (p=0.001). This finding is supported by a study conducted in Kampala, Uganda which implicated poor physical health of women as a risk factor for developing PPD.^6^ The findings in this study also implicated postpartum blues as a risk factor for developing PPD. Over half (51.8%) of the women who experienced postpartum blues within 0–2 weeks after delivery had PPD. However, a study in Nairobi, Kenya reported that postpartum blues was not associated with PPD.^39^ The method of feeding the baby was significantly associated with PPD (p= 0.003), 69.2% of the women who exclusively breast fed their babies did not have PPD, whereas 76.9% of those who bottle fed their babies had PPD. This implies that breastfeeding, being very important for infant growth and cognitive development, may in addition, be a better option for feeding a baby to avoid PPD. Having a baby of a non-desired sex, was identified as a risk factor for PPD in studies conducted in Australia^40^ and France. 41 However in this study, it was not identified as a risk factor associated with having PPD. Other studies in Kenya^6^ and Turkey^42^ identify the sex of a baby as being linked to having PPD. Reports on sex preference and development of PPD has varied from culture to culture. In China, a study indicated that giving birth to a baby girl was significant in the development of PPD^43^. Also, women who had problems during pregnancy/complications were reported in studies from Morocco,^9^ Nigeria 44 and Uganda,^6^ to be linked with developing PPD. However, in this study, there was no association between having problems/complication during pregnancy and having PPD. In this study intimate partner violence was significantly associated with PPD (p<0.001). Similarly, various studies attributed physical abuse by a partner as a risk factor associated with PPD. 30,45 On the contrary, a study conducted in India did not find any significant relationship between PPD and physical abuse.^46^ This study also reported not having help with caring for self and the baby after birth (p<0.001) and having unsupportive husbands (p<0.001) as risk factors for developing PPD. Similarly, a study in Turkey recognized lack of social support as a predictor for PPD.^35^ This implies that the role of having a good support system can never be over emphasized. Studies in both developed and developing countries have depicted the importance of having good social support in decreasing the risk of having PPD.^47^,^48^ Having postpartum blues, not having help after childbirth, experiencing intimate partner violence and having an unsupportive partner were identified as predictors of PPD, as corroborated by other studies. A meta-analysis identified prenatal depression, low self-esteem, childcare stress, prenatal anxiety, life stress, social support, poor marital relationship, history of previous depression, infant temperament, maternity blues, marital status, socioeconomic status, and unplanned/unwanted pregnancy as predictors of PPD.^49^ A systematic review also found a few biological and psychosocial predictive factors of PPD, the strongest predictors being severe life events, some forms of chronic strain, relationship quality, and support from partner and mother.^50^ Another systematic review found the current best predictors to be clinical assessments for psychiatric history and adverse life events, highlighting the need for increased depression screening across the perinatal period.^51^

## Conclusion

This study revealed a high prevalence of PPD among women attending PHCs at six weeks post-delivery. Among the factors associated with PPD, experiencing postpartum blues, not getting help with child care, intimate partner violence, and having an unsupportive partner were identified as predictors of PPD. Adequate social systems that provide support for women before and after delivery should be instituted. Simple screening methods applied early in the postpartum period should be made routine for all women attending postnatal clinics and even up to a year or two after delivery.

## References

[1] Beck CT, Records K, Rice M (2006). Further development of the postpartum depression predictors inventory-revised. J Obstet Gynecol Neonatal Nurs.

[2] Katon W, Russo J, Von-Korff M, Lin E, Simon G, Bush T, Ludman E, Walker E (2002). Long-term effects of a collaborative care intervention in persistently depressed primary care patients. J. Gen. Intern. Med.

[3] Robertson E, Celasun N, Stewart DE, Stewart DE, Robertson E, Dennis CL, Grace SL, Wallington T (2003). Risk factors for postpartum depression. Postpartum depression: Literature review of risk factors and interventions.

[4] Rai S, Pathak A, Sharma I (2015). Postpartum psychiatric disorders: Early diagnosis and management. Indian Journal of Psychiatry.

[5] World Health Organization (WHO) (2003). Managing complications in pregnancy and childbirth: A guide for midwives and doctors.

[6] Halbreich U, karkun S (2006). Cross-cultural and social diversity of prevalence of postpartum depression and depressive symptoms. Journal of Affective Disorders.

[7] Nakku J, Nakasi G, Mirembe F (2006). Postpartum major depression at six weeks in primary health care: prevalence and associated factors. African Health Sciences.

[8] Weobong B, Ten-Asbroek A, Soremekun S, Danso S, Owusu-Agyei S (2015). Determinants of postnatal depression in rural Ghana: findings from the done population-based cohort study. Depression and Anxiety.

[9] Adama N, Foumane P, Olen J, Dohbit J, Meka E (2015). Prevalence and Risk Factors of Postpartum Depression in Yaounde, Cameroon. Open Journal of Obstetrics and Gynecology.

[10] Agoub M, Moussaoui D, Battas O (2005). Prevalence of postpartum depression in a Moroccan sample. Archives of Women's Mental Health.

[11] Adewuya OA, Eegunranti A, Lawal A (2005). Prevalence of postnatal depression in Western Nigerian women: a controlled study. International Journal of Psychiatry in Clinical Practice.

[12] Adewuya AO (2006). Early postpartum mood as a risk factor for postnatal depression in Nigerian women. Am J Psychiatry.

[13] Abasiubong F, Bassey EA, Ekott JU (2008). Postpartum depression among women in Uyo, Akwa-Ibom State. Niger J Psychiatry.

[14] Uwakwe R (2003). Affective (depressive) morbidity in puerperal Nigerian women: Validation of the Edinburgh Postnatal Scale. Acta Psychiatr Scand.

[15] Obindo TJ (2014). Prevalence and correlates of postpartum depression in a teaching hospital in Nigeria. Highland Medical Research Journal.

[16] Tungchama F, Obindo J, Armiyau A, Maigari Y, Davou F, Goar S (2018). Prevalence and sociodemographic correlates of postpartum depression among women attending Postnatal and/or Children's Welfare Clinics in a Tertiary Hospital, Jos, Nigeria. Sahel Med J.

[17] Hamdan A, Tamim H (2011). Psychosocial risk and protective factors for postpartum depression in the United Arab Emirates. Archives of Women's Mental Health.

[18] Haque A, Namavar A, Breene K (2015). Prevalence and risk factors of Postpartum Depression in Middle Eastern/Arab Women. Journal of Muslim Mental Health.

[19] Najafi K, Zarrabi H, Shirazi M, Avakh F, Nazifi F (2007). Prevalence of postpartum depression in a group of women delivering at a hospital in Rasht city, Iran. Journal of Pakistan Psychiatric Society.

[20] Chaaya M, Campbell O, El-Kak F, Shaar D, Harb H (2002). Postpartum depression: prevalence and determinants in Lebanon. Archives of Women's Mental Health.

[21] Peindl O, Kathleen S, Katherine L, Wisner, Barbara H (2004). Identifying Depression in the First Postpartum Year: Guidelines for screening and referral. Journal of Affective Disorders.

[22] Msiqwa T (2010). Prevalence of depressive symptoms and risk factors among postpartum mothers at Sinza and Magomeni health in Kinondoni Municipal- Dar Es Salaam, Tanzania.

[23] Joel AA, Olayinka OA, Rejuaro FM, Yusuf A, Chibuike O (2016). Knowledge of Postpartum Depression and its Associated Risk Factors Among Nurse-Midwives in a Nigerian Tertiary Hospital. Sierra Leone J Biomed Res.

[24] Cochran WG (1963). Sampling Techniques.

[25] Cox J, Holden J, Sagovsky R (1987). Detection of postnatal depression: development of the 10-item Edinburgh postnatal depression scale. British Journal of Psychiatry.

[26] Wachs TD, Black MM, Eagle PL (2009). Maternal depression: A global threat to children's health, development, and behaviour and human rights. Child Development Perspectives.

[27] Cooper PJ, Tomlinson M, Swartz L, Woolgar M, Murray L, Molteno C (1999). Postpartum depression and the mother-infant relationship in a South African peri-urban settlement. Br J Psychiatry.

[28] January J, Burns J, Chimbari M (2017). Primary care screening and risk factors for postnatal depression in Zimbabwe: A scoping review of literature. J Psychol Africa.

[29] Ukaegbe CI, Iteke OC, Bakare MO, Agbata AT (2012). Postpartum Depression among Igbo Women in an Urban Mission Hospital, South East Nigeria. EMJ.

[30] Owoeye OA, Aina OF, Morakinyo O (2006). Risk factors of postpartum depression and EPDS scores in a group of Nigerian Women. Trop Doct.

[31] Chinawa JM, Israel O, Ndu IK, Ezugwu EC, Aniwada EC, Chinawa AT (2016). Postpartum depression among mothers as seen in hospitals in Enugu, South-East Nigeria: an undocumented issue. Pan Afr Med J.

[32] Ahmed HM, Alalaf SK, Al-Tawil NG (2012). Screening for postpartum depression using Kurdish version of Edinburgh postnatal depression scale. Arch Gynecol Obstet.

[33] Norhayati MN, Nik Hazlina NH, Asrenee AR, Wan Emilin WMA (2015). Magnitude and risk factors for postpartum symptoms: A literature review. J Affect Disord.

[34] Lanes A, Kuk JL, Tamim H (2011). Prevalence and characteristics of Postpartum Depression symptomatology among Canadian women: a cross-sectional study. BMC Public Health.

[35] Green K, Broome H, Mirabella J (2006). Postnatal depression among mothers in the United Arab Emirates: Socio-cultural and physical factors. Psychol Health Med.

[36] Gaillard A, Le Strat Y, Mandelbrot L, Keïta H, Dubertret C (2014). Predictors of postpartum depression: Prospective study of 264 women followed during pregnancy and postpartum. Psychiatry Res.

[37] Goker A, Yanikkerem E, Demet MM, Dikayak S, Yildirim Y, Koyuncu FM (2012). Postpartum depression: is mode of delivery a risk factor?. ISRN Obstet Gynecol.

[38] Koutra K, Vassilaki M, Georgiou V, Koutis A, Bitsios P, Chatzi L (2014). Antenatal maternal mental health as determinant of postpartum depression in a population based mother-child cohort (Rhea Study) in Crete, Greece. Soc Psychiatry Psychiatr Epidemiol.

[39] Oates J, Cox S, Neema P, Asten N, Glangeaud-Freudenthal B, Figueiredo LL (2004). Postnatal depression across countries and cultures: a qualitative study. The British Journal of Psychiatry.

[40] Boyce P, Hickey A (2005). Psychosocial risk factors to major depression after childbirth. Soc Psychiatry Psychiatr Epidemiol.

[41] De-Tychey C, Briançon S, Lighezzolo J, Spitz E, Kabuth B, de Luigi V (2007). Quality of life, postnatal depression and baby gender. J Clin Nurs.

[42] Kim Y-K, Hur J-W, Kim K-H, Oh K-S, Shin Y-C (2008). Prediction of postpartum depression by socio-demographic, obstetric and psychological factors: A prospective study. Psychiatry Clin Neurosci.

[43] Beck CT (2006). Postpartum Depression: It isn't just the blues. American Journal of Nursing.

[44] Abiodun OA (2006). Postnatal depression in Primary Care Population in Nigeria. Gen Hosp Psych.

[45] Leahy-Warren P, McCarthy G, Corcoran P (2012). First-time mothers: social support, maternal parental self-efficacy and postnatal depression. J Clin Nurs.

[46] Savarimuthu RJS, Ezhilarasu P, Charles H, Antonisamy B, Kurian S, Jacob KS (2010). Postpartum Depression in the Community: A Qualitative study from rural South India. Int J Soc Psychiatry.

[47] Sword W, Kurtz Landy C, Thabane L, Watt S, Krueger P, Farine D (2011). Is mode of delivery associated with postpartum depression at 6 weeks: a prospective cohort study. BJOG An Int J Obstet Gynaecol.

[48] Lanes A, Kuk JL, Tamim H (2011). Prevalence and characteristics of Postpartum Depression symptomatology among Canadian women: a cross-sectional study. BMC Public Health.

[49] Beck CT (2001). Predictors of postpartum depression: an update. Nursing Research.

[50] Yim IS, Tanner Stapleton LR, Guardino CM, Hahn-Holbrook J, Dunkel Schetter C (2015). Biological and psychosocial predictors of postpartum depression: systematic review and call for integration. Annu Rev Clin Psychol.

[51] Guintivano J, Manuck T, Meltzer-Brody S (2018). Predictors of Postpartum Depression: A Comprehensive Review of the Last Decade of Evidence. Clinical Obstetrics and Gynecology.

